# What do archaeal and eukaryotic histidine kinases sense?

**DOI:** 10.12688/f1000research.20094.1

**Published:** 2019-12-27

**Authors:** Nicolas Papon, Ann M. Stock

**Affiliations:** 1 Groupe d’Etude des Interactions Hôte-Pathogène (GEIHP, EA 3142), SFR ICAT 4208, UNIV Angers, UNIV Brest, Angers, France; 2 Department of Biochemistry and Molecular Biology, Center for Advanced Biotechnology and Medicine, Rutgers-Robert Wood Johnson Medical School, Piscataway, NJ, 08854, USA

**Keywords:** two-component system, histidine kinase, sensor, evolution, signal transduction, phosphorylation

## Abstract

Signal transduction systems configured around a core phosphotransfer step between a histidine kinase and a cognate response regulator protein occur in organisms from all domains of life. These systems, termed two-component systems, constitute the majority of multi-component signaling pathways in Bacteria but are less prevalent in Archaea and Eukarya. The core signaling domains are modular, allowing versatility in configuration of components into single-step phosphotransfer and multi-step phosphorelay pathways, the former being predominant in bacteria and the latter in eukaryotes. Two-component systems regulate key cellular regulatory processes that provide adaptive responses to environmental stimuli and are of interest for the development of antimicrobial therapeutics, biotechnology applications, and biosensor engineering. In bacteria, two-component systems have been found to mediate responses to an extremely broad array of extracellular and intracellular chemical and physical stimuli, whereas in archaea and eukaryotes, the use of two-component systems is more limited. This review summarizes recent advances in exploring the repertoire of sensor histidine kinases in the Archaea and Eukarya domains of life.

## Introduction

Protein phosphorylation is one of the most extensively used modifications in signal transduction pathways in both prokaryotic and eukaryotic cells. Prominent families of enzymes that perform protein phosphorylation encompass serine/threonine kinases, tyrosine kinases, and histidine kinases (HKs). Although HKs dominate prokaryotic signaling pathways, they are less prevalent in eukaryotes ^1, 2^. A distinct class of mammalian HKs, specifically nucleoside diphosphate kinases, function together with associated phosphatases to catalyze reversible histidine phosphorylation of proteins, and the roles of such modifications in cellular regulation are beginning to be uncovered ^3– 7^. However, the large family of HKs that is prevalent in prokaryotes is absent from animals. Historically, a small number of eukaryotic HKs have been studied in plants, yeasts, filamentous fungi, and slime molds. Recent studies have expanded the characterization of HKs in other eukaryotic lineages and archaea, allowing a broader assessment of the types of signaling systems mediated by HKs and their phylogenetic distribution and evolution. HKs are central to regulatory systems that impact agriculture, the environment, and both beneficial and pathogenic interactions of microbes with humans and other animals. Their great diversity, versatility, and broad distribution, as well as the specificity of HK communication with cognate downstream components, make them attractive targets for therapeutics ^8– 12^ and biotechnological interventions ^13– 16^ and also as building blocks for engineered biosensor systems ^17– 21^.

**Figure 1.**
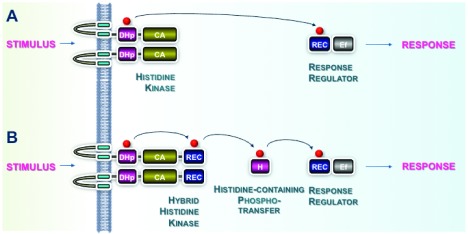
Two-component system phosphotransfer schemes. ( **A**) A typical phosphotransfer pathway, as is usually found in prokaryotes. The perception of a stimulus by extracytoplasmic domains of the histidine kinase (HK) regulates its activities. The HK autophosphorylates at a conserved histidine residue (H) using ATP bound to the catalytic ATPase domain (containing conserved motifs N, G1, F, and G2). The phosphoryl group (P) is transferred to a conserved aspartate residue (D) located within the cognate response regulator (RR). ( **B**) An example of a multi-step phosphorelay, as often occurs in eukaryotes. The HK is termed “hybrid” because an additional aspartate-containing domain is fused to the ATPase domain. The phosphorelay involves multiple phosphoryl transfer steps. The first is an intramolecular transfer between the conserved histidine (H) and a conserved aspartate residue (D) located within the C terminus of the sensor HK. Subsequently, the phosphoryl group is transferred to a histidine-containing phosphotransfer protein and finally to a cognate RR. Conserved domains of the two-component system (TCS) proteins are shown in green, gold, and blue. Variable sensor domains of the HK and effector domains (Ef) of the RR that adapt the systems to a wide range of input stimuli and output responses are shown in gray.

HKs occur primarily within pathways designated as “two-component systems” (TCSs) ^22, 23^ ( Figure 1). TCSs correspond to cell signaling circuitries that permit organisms, either unicellular or multicellular, to sense and respond to a broad palette of environmental changes. From a mechanistic perspective, these transduction pathways rely on the sequential transfer of a phosphoryl group on conserved histidine or aspartate residues (or both) located in several families of proteins. In prokaryotes, TCSs are usually restricted to communication between two functional modules (that is, phosphoryl transfer between HKs and response regulators [RRs]) ( Figure 1A). In canonical prokaryotic systems, the perception of a stimulus regulates the opposing autophosphorylation and phosphatase activities of the HK, which thus acts as a primary sensor. The phosphoryl group is transferred to a RR that effects the response. In many prokaryotic TCSs (~65%), the RR is a transcription factor that directly regulates the expression of a set of genes required for an adaptive response to the stimulus ^24, 25^.

In contrast, classic eukaryotic TCSs usually involve more complex multi-step phosphorelays ^26, 27^ but, as in prokaryotes, also begin by the perception of an input stimulus by a sensor HK, specifically a “hybrid” HK ( Figure 1B). Signal perception modulates autophosphorylation of the HK on a conserved histidine residue prior to transfer to a conserved aspartate residue in a C-terminal domain of the HK. The phosphoryl group is then transmitted to a conserved histidine residue of a small shuttle protein of about 150 amino acid residues (histidine-containing phosphotransfer protein, HPt; Pfam ^28^ ID PF01627) and finally to a conserved aspartate residue of a protein belonging to the RR family. The phosphorylation state of this RR orchestrates subsequent molecular events underlying the response to the input signal, either by directly regulating transcription or by interfacing with other conventional eukaryotic signaling strategies such as mitogen-activated protein kinase cascades or cAMP signaling that control the output response. As a result of specific evolutionary paths in which the various eukaryotic lineages have engaged, the canonical TCS pathway described above appears to have degenerated in several clades in which these cell signaling systems have been described.

**Figure 2.**
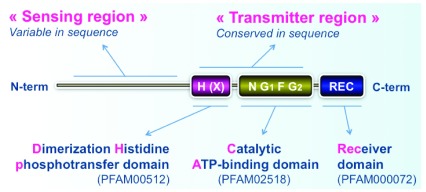
Canonical structure of histidine kinases (HKs). HKs are composed of a set of variable and conserved domains. The first region corresponds to a highly variable, typically N-terminal sequence that determines which stimulus is perceived by the HK. This region is referred to as the “sensing domain”. The central “transmitter region” is composed of two conserved domains: a dimerization histidine phosphotransfer (DHp) domain (His kinase A, HisKA; Pfam ID PF00512, or other subfamily such as HisKA_2, HisKa_3) and a catalytic ATP-binding (CA) domain (histidine kinase-like ATPase catalytic, HATPase_c; Pfam ID PF02518). The DHp domain includes an H-box, usually containing the phosphorylatable histidine, and an X-box. The CA subdomain includes four distinct sequence motifs: the N-, G1-, F-, and G2-boxes. In contrast to prokaryotic HKs, most eukaryotic HKs contain an additional C-terminal RR receiver (REC) domain (Response_reg; Pfam ID PF00072) that includes a phosphorylatable aspartate residue. Thus, eukaryotic HKs are generally called “hybrid HKs” ^26^.

The canonical structure of HKs is composed of a set of variable and conserved domains ^29^ ( Figure 2) that couple the sensing of a wide range of chemical or physical stimuli to the phosphotransfer pathway. Here, we review recent advances in the characterization of HKs in archaea and eukaryotes with an emphasis on what they sense and what the main output processes that this prominent family of sensors regulates are. Some emerging trends dealing with their distribution among the various lineages and their evolution are also considered.

## HKs in Archaea

TCSs are relatively rare in archaea and are not uniformly distributed across archaeal phyla ^30– 32^. The majority of archaeal TCSs have been identified in *Euryarchaeota* and *Thaumarchaeota*, but the possibility exists that greater distribution will be revealed as more archaeal genomes are sequenced. Several notable features differ between archaeal and bacterial TCSs ^32^. Archaeal genomes typically contain fewer TCSs and typically have an HK-to-RR ratio greater than 1, suggesting that multiple HKs might feed into a single RR or that HKs might be paired with alternative downstream components. Unlike bacterial HKs that are mostly transmembrane proteins (estimated at 73 to 88%), 62% of archaeal HKs lack identifiable transmembrane regions and are presumed to be cytoplasmic. Interestingly, previous analyses of bacterial and archaeal chemoreceptors have shown a similar bias for cytoplasmic sensing in archaea ^33^. Correspondingly, although extracellular Cache—calcium channels and chemotaxis receptors, also previously identified as PAS (period circadian protein-Aryl hydrocarbon receptor nuclear translocator protein-single-minded protein), PAS-like, PhoQ-DcuS-CitA (PDC), PDC-like, and PDC/PAS ^34– 36^ domains—are the most abundant sensor domains in bacterial HKs, intracellular PAS and GAF (cGMP-specific phosphodiesterases-adenylyl cyclases-FhlA) domains are predominant in archaeal HKs, and 72% of them contain one or more PAS or GAF domains (or both) ^32^. In addition to Cache domains, less populated sensor domain families include MEDS (methanogen/methylotroph, DcmR sensory domain; Pfam ID PF14417), PocR (Pfam ID PF10114, HisKA_7TM (Pfam ID PF16927), and HisKA_4TM (Pfam ID PF16926), the latter being distinct to haloarchaea.

Other than chemotaxis ^37, 38^ and phototaxis ^39, 40^ systems, few archaeal TCS pathways have been characterized. Two recently studied systems, similar to conventional TCSs, are the LtrK/LtrR TCS that mediates temperature-dependent gene regulation in an Antarctic methanogen ^41^ and a TCS comprised of HK FilI and RRs FilR1 and FilR2 that regulates transcription of methanogenesis genes in response to unknown stimuli in *Methanosaeta harundinacea* ^42^. Two less conventional HKs, each containing multiple PAS and GAF domains, have recently been characterized from *Methanosarcina acetivorans* ^43, 44^. MsmS is a heme-based redox/dimethyl sulfide sensor, and RdmS is a thiol-based redox sensor; both regulate genes involved in methyl sulfide metabolism. Autophosphorylation of both HKs is redox-dependent, although neither contains a phosphorylatable histidine and MsmS phosphorylation has been shown to occur at tyrosine. Furthermore, the identified downstream regulators lack RR receiver (REC) domains, indicating that these HKs function in signaling systems distinct from TCSs.

The outputs of archaeal TCSs are currently as unexplored as the inputs. However, unlike archaeal HK sensor domains that belong to families common to bacterial counterparts such as PAS and GAF, albeit with different prevalence, archaeal RR effector domains do not correspond to the major families found in bacterial RRs ^32^. Most notable is the low abundance of recognizable DNA-binding domains (6%). The fraction of archaeal RRs consisting solely of REC domains (39%) is almost double that observed in bacteria. Other major families include dimerization histidine phosphotransfer (DHp) domains (that is, HisKA), PAS _*n*_, GAF, PAS-GAF, chemotaxis CheB, HalX (with a predicted helix-turn-helix, possibly DNA-binding), and various enzyme domains as well as several novel domain families.

## HKs in Eukarya

Historically, plants were the first eukaryotic kingdom in which HKs were identified and functions of plant HKs have been informed primarily in the last decades by descriptions of TCSs in the model plant *Arabidopsis thaliana* ^45– 48^. Along with these pioneering works in plants, some groups of HKs were progressively characterized in other eukaryotic lineages such as Amoebozoa (mainly the slime mold *Dictyostelium discoideum*) ^49– 52^ and Fungi (mainly the yeasts *Saccharomyces cerevisiae*, *Candida albicans*, and *Cryptococcus neoformans* and the filamentous fungi *Neurospora crassa* and *Aspergillus fumigatus*) ^53, 54^. To date, what the biological roles in the remaining eukaryotic phyla are and what HKs sense are still largely obscure ^55^. In the following section, we will briefly describe major groups of eukaryotic HKs, notably their structures, phylogenetic distribution, and their roles in hormone perception, stress adaptation, and developmental programs.

The first group of eukaryotic HKs characterized is now well documented to be dedicated to the perception of and response to the plant hormone (phytohormone) ethylene ( Table 1) ^56^. Ethylene is a gas that regulates many aspects of plant development such as seed germination, leaf senescence, and fruit ripening but also orchestrates plant defenses to pathogens (viruses, protists, bacteria, fungi, worms, and insects) ^57^. From a structural point of view, it is important to highlight that ethylene sensing through HK ethylene receptors (ETRs) occurs by the interaction of the gaseous molecule with the ethylene-binding domain (EtBD) located at the N terminus of the receptors. The EtBD consists of three hydrophobic transmembrane helices (indicated by three asterisks in Table 1) containing seven conserved amino acids required for ethylene binding ^58^. For a long time, typical ETRs were believed to be restricted to land plants and cyanobacteria ^59^. Surprisingly, these considerations have now been called into question through the identification in recent years of genes encoding ETR homologs in many other eukaryotic lineages, including green and brown algae, free-living amoebae, photosynthetic diatoms, zooxanthellae that are symbiotically associated with coral reefs, early diverging fungi, filamentous marine protists ( *Labyrinthulomycetes*), and even the unicellular model animal ancestor *Capsaspora owczarzaki* ^60– 64^. Interestingly, an EtDB coupled to a phytochrome domain has recently been identified in a cyanobacterial HK, integrating both light and ethylene responses ^65, 66^. These discoveries thus provide progressively strong arguments leading to the hypothesis that ethylene, more than strictly a plant hormone, would undoubtedly be one of the oldest molecules of intra- and inter-species communication that appeared on Earth, orchestrating not only developmental programs but also biotic interactions between many organisms ^67^.

**Table 1.** Some important groups of eukaryotic histidine kinases (HKs), their known input signals, and their output responses.

HK group	Structure	Presence in eukaryotes	Input signal	Output response	References
**Ethylene** **receptors**	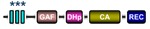	Plants, Algae, Fungi, Amoebae,	Ethylene	Plants: seed germination, leaf senescence, fruit ripening, defenses to pathogens	Ju *et al*., 2015 ^60^ Hérivaux *et al*., 2017 ^61^ Kabbara *et al*., 2019 ^64^
**CHASE-HK**	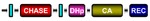	Plants, Algae, Fungi, Amoebae,	Cytokinins	Plants: cell division, embryogenesis, vascular tissue development	Kaltenegger *et al*., 2018 ^74^ Hérivaux *et al*., 2017 ^61^
**AHK1/Fungal** **group VI**	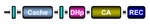	Plants, Algae, Fungi	Osmostress Oxidant stress	Plants: seed desiccation, vegetative stress tolerances Fungi: osmotic and oxidant adaptation	Defosse *et al*., 2015 ^53^ Nongpiur *et al*., 2019 ^87^
**Phytochromes**	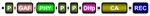	Plants, Algae, Fungi Amoebae,	Red/far red light	Plants: phototropism Fungi: vegetative growth, sexual reproduction	Rensing *et al*., 2016 ^88^ Yu and Fischer, 2019 ^89^
**CKI1**	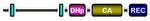	Plants	Cytokinins?	Development of female gametophyte	Yuan *et al*., 2016 ^92^ Liu *et al*., 2017 ^93^ Yuan *et al*., 2018 ^94^
**CKI2/AHK5**	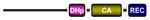	Plants	?	Stress-induced stomatal closure, salt sensitivity, and resistance against microbial infection	Pham *et al*., 2012 ^95^ Mira-Rodado *et al*., 2012 ^96^ Bauer *et al*., 2013 ^97^
**Fungal group III**	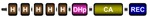	Fungi, Amoebae	Osmostress	Fungi: oxidant adaptation, development, virulence	Defosse *et al*., 2015 ^53^ Hérivaux *et al*., 2016 ^54^ Kabbara *et al.*, 2019
**Fungal group X**	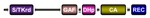	Fungi, Algae, Amoebae	Oxidant stress ?	Fungi: oxidant adaptation, development, virulence	Defosse *et al*., 2015 ^53^ Hérivaux *et al*., 2016 ^54^ Kabbara *et al.*, 2019 ^64^

KEY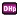:Dimerization histidine phosphotransfer domain 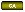:Catalytic ATP-binding domain 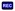:Rec eiver domain 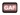: cGMP-specific phosphodiesterases- Adenylyl cyclases- FhlA domain 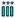:Ethylene Binding Domain 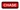:Cyclases/ Histidine kinases Associated Sensing Extracellular 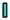:Trans membrane Region 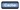:Calcium channels and Chemotaxis receptors domain 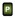:Period circadian protein- Aryl hydrocarbon receptor nuclear translocator protein- Single-minded protein 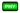:Phytochrome domain 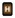:Histidine kinases- Adenylate cyclases- Methyl accepting proteins and Phosphatases 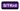:Serine/ Threonine kinase related domain

A second well-known eukaryotic HK group encompasses CHASE (cyclases/histidine kinases associated sensing extracellular) ^68, 69^ domain-containing HKs (CHASE-HKs) ( Table 1). To date, most of the members belonging to this group have been characterized in plants as cytokinin receptors ^62, 70^. Cytokinins correspond to another family of prominent phytohormones involved in many developmental processes in plants, including cell division, embryogenesis, vascular tissue development, and root architecture ^71^. The hormone is perceived by this type of transmembrane HK through the N-terminal region that comprises an extracellular loop ^70, 72^. More precisely, some crucial residues have been identified within the CHASE sequence to be essential for the binding of the hormones ^70, 72, 73^. As initially postulated for ethylene, cytokinin signal transduction pathways were presumed to be found exclusively in plants ^74^. However, these hormones have recently been the subject of very interesting advances that suggest a broader occurrence in the tree of life, notably in eubacteria ^75^ and eukaryotic microorganisms ^62^. It has been experimentally shown that a bacterial CHASE-HK senses cytokinin, highlighting the importance of HK-cytokinin interactions in inter-kingdom communication ^75^. In addition, several genes encoding CHASE-HKs were unearthed in the past two years by browsing the genomes of various non-plant eukaryotic clades ^61– 64^. These include, for example, some early diverging fungi, brown algae, and diatoms. Although these latter homologs have not been functionally characterized to date, the phylogenetic distribution of CHASE-HKs within the various eukaryotic clades suggests an unexpected and broad involvement of cytokinins and their HK receptors in the regulation of various physiological processes of eukaryotic organisms and interspecies interactions ^62^.

A third group of eukaryotic HKs involves transmembrane receptors that have been reported to be involved mostly in osmosensing ^47, 49^. The first members of this group were characterized at the beginning of the 1990s in yeast (referred to as fungal group VI of HKs) and a few years later in plants (referred to as AHK1) ( Table 1). In *Saccharomyces* cells, these receptors are known to allow the yeast to respond and adapt to osmotic and (to a lesser extent) oxidant stresses ^53, 76^. In *Arabidopsis*, the TCS controlled by AHK1 was reported to perceive water stress and to initiate histidine-to-aspartate phosphotransfer circuitry for seed desiccation and vegetative stress tolerances ^47, 77– 87^. From a structural perspective, these fungal and plant osmosensors include two large hydrophobic transmembrane helices that border a roughly 300–amino acid extracellular loop predicted to fold mostly into large helices and small sheets, recently identified as a Cache domain ^36^. Pioneering studies on this type of receptor demonstrated that expression of plant AHK1 genes can complement the lack of the unique and essential fungal group VI HK gene in yeast, indicating that plant and yeast putative osmosensors have common functional features and origins ^47, 78^. Interestingly, however, recent genome-wide analyses suggested that these structural and functional similarities rely on an evolutionary convergence process rather than a common archetypal system inherited in both fungi and plants ^64^.

Another well-known group of HK-type receptors widely found in many clades of the eukaryotic domain is phytochromes ^88, 89^. Phytochromes consist of photo-switchable red/far-red photoreceptors that likely evolved in cyanobacteria prior to being transferred to some eukaryotic lineages ^90, 91^ ( Table 1). In both plants and fungi, they have been demonstrated to be involved in a wide range of physiological processes ^88, 89^. Importantly, phytochrome sequences from early diverging plants, as fungal phytochromes, display all conserved amino acid residues for HK activity. In contrast, phytochromes from higher plants commonly contain HK-like domains that instead display Ser/Thr kinase activity, suggesting a structural evolution of these receptors in flowering plants toward other non-TCS output domains ^98^. Some recent genome-wide analyses—and, in some cases, functional characterization studies—demonstrated that phytochromes also occur in green and brown algae, diatoms, and amoebae ^63^.

There are also other important groups of eukaryotic HKs that have been deeply studied in recent years. These include, for instance, two plant-specific groups: CKI1, which is involved in female gametophyte development ^92– 94^, and the CKI2/AHK5, which was recently shown to govern the stress-induced stomatal closure, salt sensitivity, and resistance against microbial infection in *Arabidopsis* ^95– 97^ ( Table 1). Their precise input signals remain unknown. Finally, recent classification of HKs in fungi revealed that these sensing proteins could be categorized into 16 groups ^64^; among these, groups III and X seem to play important roles in stress adaptation, morphogenesis, and virulence ^53, 54^ ( Table 1).

## Conclusions

Sequence information is available for a large number of HK sensor domains, enabling identification of abundantly populated fold families. A relatively small number of common sensor domains appear across all domains of life, although their abundance is strongly skewed in different organisms. Unfortunately, sequences and fold families often provide little information about ligands or physical stimuli (or both) detected by individual domains if experiences with bacterial HKs are generalizable.

Although studies of bacterial sensing have focused on a small number of structural folds such as Cache and four-helix bundle domains ^99^, it is sobering to note that the largest class of bacterial HKs, the prototypical HKs with periplasmic sensing domains, contain 50 to 300 residue-sensing domains that bear no sequence similarity to domains of known folds or functions ^100^. Even when folds are identifiable, a similar fold can bind many different types of ligands and conversely the same ligand can be bound by domains of different folds in different proteins. Furthermore, even in extensively studied bacterial TCSs where a general stimulus such as cell wall stress is known, the exact molecule or physical parameter sensed by the HK often remains undetermined. Even for some extensively characterized *Escherichia coli* TCSs, where investigative tools include robust genetics and atomic-resolution three-dimensional structures, specific stimuli remain unidentified. The problem of identifying input stimuli becomes even more complex when multiple sensor kinases or heterodimeric kinases (or both) are integrated into pathways such as the LadS/GacS/RetS/PA1611 system in which interactions among four HKs regulate biofilm formation in *Pseudomonas aeruginosa* ^101– 103^. In this system, the phosphorelay between HKs LadS and GacS is inhibited by RetS-GacS heterodimer formation which is further regulated by interactions between RetS and hybrid HK PA1611.

Remarkably, in plants, most TCSs are characterized with regard to the input stimuli and output responses. This is not true for TCSs of fungi and amoeba, and even less is known about TCSs of archaea. Although genomic analyses are a powerful tool for initial identification, experimental strategies will likely be required to drive discovery of system inputs. Recently, a previously uncharacterized *Shewanella oneidensis* HK was found to sense pH in a high-throughput screen of seven different *S. oneidensis* TCSs, using engineered RRs with *S. oneidensis* REC domains linked to a heterologous DNA-binding domain paired with a cognate reporter gene in *E. coli* ^16^. To the extent that heterologous proteins are functional, synthetic biology approaches such as this promise to provide a powerful strategy for identification of sensory inputs.

Two-component signaling provides a versatile molecular mechanism for stimulus-response coupling, and TCS protein architecture potentially allows an almost limitless range of inputs and outputs. Indeed, enough of the more than 300,000 TCSs ^28^ have been characterized to conclude that bacteria use His-Asp phosphotransfer for almost all categories of signal transduction needs. This does not appear to occur in other domains of life where regulatory systems involving Ser/Thr and Tyr phosphorylation abound. Given the great diversity of sensing and responses in bacterial TCSs, it is curious that archaeal and eukaryotic TCSs appear to have been evolved for a narrower range of functions.

## Abbreviations

Cache, calcium channels and chemotaxis receptors; CHASE, cyclases/histidine kinases associated sensing extracellular; EtBD, ethylene-binding domain; ETR, ethylene receptor; GAF, cGMP-specific phosphodiesterases-adenylyl cyclases-FhlA; HK, histidine kinase; PAS, period circadian protein-Aryl hydrocarbon receptor nuclear translocator protein-single-minded protein; PDC, PhoQ-DcuS-CitA; REC, receiver; RR, response regulator; TCS, two-component system
